# Prevalence and Management of Severe Hand, Foot, and Mouth Disease in Xiangyang, China, From 2008 to 2013

**DOI:** 10.3389/fped.2020.00323

**Published:** 2020-07-10

**Authors:** Jian Liu, Jing Qi

**Affiliations:** ^1^Department of Pediatrics, The Second School of Clinical Medicine, Affiliated Baoan Hospital of Shenzhen, Southern Medical University, Guangzhou, China; ^2^Department of Pediatrics, The Second Affiliated Hospital of Shenzhen University, Shenzhen, China; ^3^Department of Neurology, Affiliated Baoan Hospital of Shenzhen, Southern Medical University, Guangzhou, China

**Keywords:** hand, foot, mouth disease, human enterovirus 71, coxsackievirus A16, lung-protective ventilation, contagious disease

## Abstract

Therapeutic strategies for severe hand, foot, and mouth disease (HFMD) are currently either inconsequent or deficient in evidence. We retrospectively surveyed HFMD outbreaks in Xiangyang from June 2008 to December 2013. HFMD is staged from I to V according to clinical severity. Severe HFMD is defined as a case involving the central nervous system (CNS). We analyzed risk factors for fatality of severe cases and compared the efficiency and outcome of some therapies by binary logistic regression. The overall HFMD cases included 637 (1.26%) severe cases and 38 fatalities (0.075%). Analyses indicate that age (<3 years), enterovirus 71 (+), autonomic nervous system dysregulation, pulmonary edema/hemorrhage, C-reactive protein (CRP) (>40 mg/L), and cardiac troponin I (>0.04 ng/ml) are risk factors for fatality (all *P* < 0.05). Intravenous immunoglobulin (IVIG) and mechanical ventilation applied only in early stage IV significantly improved HFMD progression (both *P* < 0.05) with odds ratios of 0.24 (95% CI: 0.10–0.57) and 0.01 (95% CI: 0.00–0.10), respectively. Neither methylprednisolone nor milrinone administered in any stage made any significant difference on mortality (all *P* > 0.05). Precise recognition of the severe HFMD cases in early stage IV and prompt IVIG and mechanical ventilation application may reduce mortality. Mechanical ventilation training programs and dispatch of specialists to hospitals where there is no chance of transferring critical cases to the severe HFMD designated hospitals are two key measures to reduce fatality.

## Background

Hand, foot, and mouth disease (HFMD) is a contagious viral disease caused by more than 20 enteroviruses, including enterovirus 71 (EV71) and coxsackievirus A16 (CA16) (1, 2). It usually affects infants and children younger than 5 years. Typical clinical manifestations of HFMD include fever, herpangina, herpes, and/or maculopapular rash on palms, soles, buttocks, or limbs (3). Severe HFMD is defined as a case involving the central nervous system (CNS) (4). There is no specific treatment for the disease. Hospitalization becomes necessary when the cases get involved with uncommon neurological complications such as meningitis, brain stem encephalitis, acute flaccid paralysis, or pulmonary edema/hemorrhage. These complications can be lethal, particularly to those aged <5 years (1–4).

HFMD has been categorized as a category C notifiable disease in China since May 2, 2008. There have been many HFMD outbreaks in many cities and provinces in China in recent years (4–10). But less is known about what caused the different characteristics of epidemiology of HFMD between different areas (5, 7–10), and therapeutic strategies of severe cases are currently either inconsequent or deficient in evidence (11). Xiangyang was one of the afflicted areas where continuous outbreaks occurred yearly from 2008 to 2013. We retrospectively investigated some epidemiological characteristics of HFMD in Xiangyang, analyzed risk factors causing mortality, and compared the outcome of therapies in this study.

## Methods

### Study Population and Data Collection

Xiangyang is the second largest city of Hubei Province, China; and it administers three districts, three county-level cities, and three counties. It has a population of more than 6 million people now. The city has a subtropical monsoon continental climate with four well-demarcated seasons. Its daily temperature averages from 13°C in winter to 21°C in summer, and the annual mean temperature is 17.0°C. The floating population is large in this city.

The health authorities in Xiangyang required primary care physicians in villages or communities to register all information of HFMD cases, including medical record, family condition such as who is taking care of the child, the environment, and sanitation condition. All HFMD cases must be reported to Xiangyang Center for Disease Control and Prevention (XCDC) after being diagnosed in a clinic. Patients, once diagnosed as having severe cases, along with their medical records and information, must be instantly transferred to one of the three severe HFMD designated hospitals. We had occasionally phoned the primary care physicians or the patients' parents when the information was incomplete. We collected surveillance data on the prevalence of HFMD in Xiangyang from XCDC from June 2008 to December 2013, as well as data of severe cases and sentinel surveillance cases from the designated hospitals in the same period. The critical cases who lost their chance to be transferred to the designated hospitals and those who died before or during transfer to the designated hospitals were all investigated and undertook etiological detection. We collected variables of all severe cases and fatalities: sex, age, family condition, stage of severity on admission, laboratory test results, medicines, ventilation parameters, and outcome.

We had followed up survivors for sequelae by the end of December 2018. Primary care physicians in villages and communities were asked to supervise the discharged HFMD cases. Those cases with any neurological symptoms after discharge were ordered to return monthly to the pediatric neurology clinic of the designated hospitals.

### Clinical Staging of Hand, Foot, and Mouth Disease Cases

HFMD cases are classified into five distinct stages according to clinical severity on admission: stage I refers to cases with fever and eruption on the hands, feet, mouth, and buttocks; isolated exanthem; or herpangina. Stage II refers to those with CNS involvement, such as aseptic meningitis, encephalitis, and acute flaccid paralysis. Clinical manifestations include lethargy, sucking weakness, ease of being startled, headache, vomiting, irritability, limb tremors, and nuchal rigidity. Stage III refers to those with autonomic nervous system (ANS) dysregulation. Clinical manifestations include resting tachycardia, profuse sweating, cold extremities, and hypertension. Stage IV refers to those with tachycardia or bradycardia, hypotension, tachypnea, cyanosis, cough with pink foamy or even bloody sputum indicating frank cardiopulmonary failure, pulmonary edema or hemorrhage, frequent seizures, and severe unconsciousness; and stage V refers to those with CNS and cardiopulmonary function gradually recovered, although neurological sequelae may remain in some cases (4). Patients in stage II to V are severe cases.

### Sample Preparation and Detection

Primary hospitals diagnosed HFMD on the basis of clinical manifestations. Serum samples from suspected HFMD cases in the designated hospitals were drawn to detect IgM antibodies to EV71 with a colloidal gold rapid test kit (WJ-20, Wantai, China) and IgM antibodies to CA16 with a diagnostic ELISA kit (WQ-1096, Wantai, China). The clinical tests applied to all severe HFMD cases, fatalities, and cases for sentinel surveillance. Their throat and rectal swabs were additionally collected to extract RNA with a Viral RNA Mini Extraction Kit (#52904, Qiagen, Germany); then a OneStep RT-PCR Kit (#210212, Qiagen, Germany) was used, followed by electrophoresis to detect EV71, CA16, and general enterovirus. RT-PCR primers were as follows: enterovirus forward primer 5′-TCC GGC CCC TGA ATG CGG CTA ATC C-3′, reverse primer 5′-ACA CGG ACA CCC AAA GTA GTC GGT CC-3′; EV71 forward primer 5′-GCA GCC CAA AAG AAC TTC AC-3′, reverse primer 5′-ATT TCA GCA GCT TGG AGT GC-3′; and CoX A16 forward primer 5′-ATT GGT GCT CCC ACT ACA GC-3′, reverse primer 5′-TCA GTG TTG GCA GCT GTA GG-3′. We also performed laboratory tests such as blood cell counting, serum glucose, C-reactive protein (CRP), and cardiac troponin I (cTnI) for severe inpatient HFMD cases on admission.

### Treatment Strategies for Severe Hand, Foot, and Mouth Disease Cases

Intravenous immunoglobulin (IVIG) and anti-inflammatory agent methylprednisolone were administered to severe HFMD cases as soon as possible after diagnosis. Two different doses, recommended by valid national or provincial HFMD guidelines (4, 12), were selected alternatively according to doctors' preference: IVIG 2 g/kg iv drip singly or 1 g/kg iv drip, qd for 2 days; methylprednisolone 20 mg/kg iv drip singly or 1–2 mg/kg iv drip, qd for 2–3 days. Milrinone is a positive inotropic cardiotonic agent and vasodilator. Milrinone was administered in cases of persistent resting tachycardia, hypertension, or profuse sweating. Milrinone usage was as follows: a loading dose of 25–75 μg/kg iv and a maintenance dose of 0.25–1.0 μg/kg per minute.

There was no precise recommendation about mechanical ventilation parameters in past few years. Thus, some doctors adopted a protective-ventilation strategy (13). Positive end-expiratory pressure (PEEP) was set at 2–3 cm H_2_O above the lower inflection point on the static pressure-volume curve, a tidal volume was set <6–8 ml/kg, and peak inspiratory pressure was <25 cm H_2_O above the PEEP value. Permissive hypercapnia and pressure-limited ventilatory modes were preferable. PEEP was set temporarily at 10 cm H_2_O above the lower inflection point for 20–40 min on pulmonary edema/hemorrhage at the beginning of mechanical ventilation, when an accidental tube disconnection or extubation, after aspiration of sputum, or in cases when pulmonary edema/hemorrhage had not been controlled or had reoccurred. In contrast, high-pressure ventilation, including PEEP above 15–20 H_2_O and peak inspiratory pressure >40 cm H_2_O, or large-volume control ventilation are characteristics of common ventilation strategy, which was applied by other doctors.

### Statistical Analysis

We analyzed the risk factors causing mortality, such as sex, age, stage of severity on admission, blood cell counting, serum glucose, CRP, and cTnI by binary logistic regression, and we compared the efficiency of different doses of IVIG and methylprednisolone, milrinone, and ventilatory strategies on severe HFMD cases in different stages of severity. We performed the statistical analysis by SPSS Statistics Version 22 (IBM Inc.).

## Results

### Prevalence of Hand, Foot, and Mouth Disease

**Figure 1:**
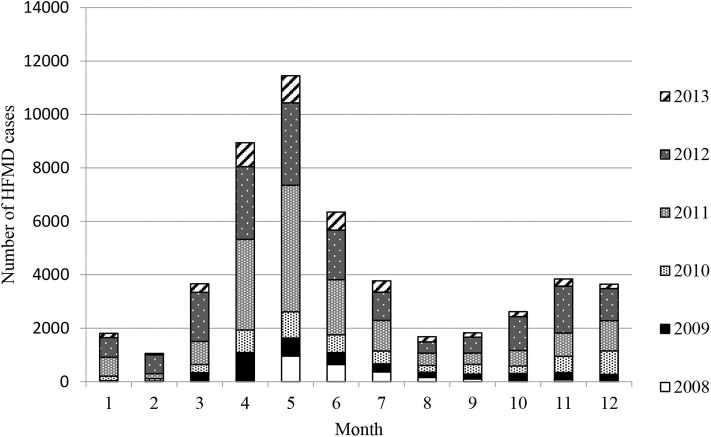
The prevalence of hand, foot, and mouth disease (HFMD) in Xiangyang from 2008 to 2013.

Epidemiological investigation revealed that the HFMD outbreaks in Xiangyang had gone through four stages in 6 years: a sudden outbreak in 2008, then quick aggravation year by year, a sharp decrease in morbidity after 2012, and then low-level tailing for several years. The HFMD outbreaks involved 50,651 cases clinically or laboratorially diagnosed, of which 637 (1.26%) were severe cases, virologically confirmed by the three designated hospitals, and 38 cases were fatal (fatality rate: 0.075%). There were only eight severe cases and two fatalities confirmed by virologic tests in first epidemic year 2008. A total of 91.14% were 5 years of age or younger, and 74.3% were 3 years of age or younger. EV71 and CA16 were responsible for 58.86 and 16.18% of all cases, 72.53 and 12.45% of the severe cases, and 94.73 and 2.63% of the deaths, respectively. Two peak incidence years had the highest EV71 positive rates: 83.70% in 2011 and 81.60% in 2012. Among the severe cases, male-to-female ratio was 1.21:1, and the median age was 1.97 years, which ranged from 28 days to 9 years. EV71 was found to be the dominating causative virus of HFMD epidemics from 2009 to 2013. There were two outbreak peaks every year: a higher peak from March to August and a lower peak from October to December (Figure 1 and Table 1).

**Table 1:** Virus detection of HFMD in Xiangyang from 2008 to 2013.

**Year**	**All cases**	**Severe cases (%)**	**Fatalities (%◦)**	Virologically confirmed cases (%)^*^	**EV71 (+) cases (%)**	**Cox A16 (+) cases (%)**	**General enterovirus (+) case (%)**
2008	2,441	37 (1.52)	2 (0.82)	10 (0.41)	5 (50.00)	1 (10.00)	4 (40.00)
2009	4,006	59 (1.47)	3 (0.75)	65 (1.62)	36 (55.38)	14 (21.54)	15 (23.08)
2010	5,903	76 (1.29)	6 (1.02)	250 (4.24)	179 (71.60)	32 (12.80)	39 (15.60)
2011	16,557	213 (1.29)	18 (1.09)	497 (3.00)	416 (83.70)	45 (9.05)	36 (7.24)
2012	17,251	217 (1.26)	8 (0.46)	2,668 (15.47)	2,177 (81.60)	201 (7.53)	290 (10.87)
2013	4,493	35 (0.78)	1 (0.22)	2,458 (54.71)	688 (27.99)	706 (28.72)	1,064 (43.29)
Total	50,651	637 (1.26)	38 (0.75)	5,948 (11.74)	3,501 (58.86)	999 (16.80)	1,448 (24.34)

* *The serologic and RT-PCR tests applied to all severe HFMD cases, fatalities, and cases for sentinel surveillance, but only eight severe cases and two fatalities were confirmed by virologic tests in first epidemic year 2008*.

*HFMD, hand, foot, and mouth disease; EV71, human enterovirus 71; Cox A16, coxsackievirus A16*.

### Analysis on Risk Factors for Fatality

We analyzed 610 severe HFMD cases including 38 fatalities for fatal risk factors by binary logistic regression, excluding the 27 cases lacking an etiologic diagnosis in 2008 (Table 2). The results indicate that age (<3 years), EV71 positivity, ANS dysregulation, pulmonary edema/hemorrhage, CRP (>40 mg/L), and cTnI (>0.04 ng/ml) are risk factors for fatality (all *P* < 0.05), but sex, hyperglycemia, leukocytosis (>13 × 10^12^/L), and thrombocytosis (>500 × 10^9^/L) are not at the 5% level. When age was categorized as <1, 1–2, 2–3, and >3 years, no further significant difference was observed for fatality (*P* > 0.05).

**Table 2:** Risk factors analysis on the fatality of severe HFMD cases by binary logistic regression.

	**Survivors**	**Fatalities**	***P***	**OR**	**95% CI**
Severe cases	572^*^	38			
Male	388	25	0.36		
<3 years	402	35	0.03	9.54	1.20–75.80
ANS dysregulation	276	32	0.02	5.77	1.40–23.85
Pulmonary edema/hemorrhage	73	36	0.00	67.59	14.12–323.45
Hyperglycemia	307	29	0.38		
EV71(+)	326	36	0.00	26.55	3.02–233.04
Leukocytosis (>13 × 10^12^/L)	376	32	0.39		
Thrombocytosis (>500 × 10^9^/L)	16	12	0.82		
CRP (>40 mg/L)	73	25	0.02	4.74	1.32–16.99
cTnI (>0.04 ng/ml)	31	29	0.00	47.69	11.51–197.57

* *Severe cases excluding 27 cases lacking an etiologic diagnosis*.

*HFMD, hand, foot, and mouth disease; ANS, autonomic nervous system; BNP, brain natriuretic peptide; CRP, C-reactive protein; cTnI, cardiac troponin I; OR, odds ratio; CI, confidence interval*.

We analyzed the medical records and family condition of severe cases and found that 23.7% of fatal cases, which is much larger than 7.3% of severe cases, were raised by grandparents (*P* < 0.05). HFMD children raised by grandparents had higher fatality.

### Efficiency Comparison Between Different Therapies

The results show that IVIG administration and mechanical ventilation are significantly relevant to the outcome of severe HFMD only in early stage IV (both *P* < 0.05), with odds ratios (ORs) of 0.23 (95% CI: 0.10–0.57) and 0.01 (95% CI: 0.00–0.10), respectively. Protective ventilation did not improve fatality as compared with common ventilation strategy (*P* > 0.05). Neither methylprednisolone nor milrinone administered in any stage made any significant differences in fatality (all *P* > 0.05). No difference in fatality was seen in our data between the large dose and low dose of IVIG or methylprednisolone administered in any stage (all *P* > 0.05) (Table 3).

**Table 3:** Efficiency comparison between different therapies and dosage administered in distinct stages.

**Therapies and dosage**	**Survivors**	**Fatalities**	***P***	**OR**	**95% CI**
In stage II on admission	356	0			
IVIG: high/low/none	139/212/11	0/0/0			
Methylprednisolone: high/low/none	252/98/6	0/0/0			
Mechanical ventilation: yes/none	0/0	0/0			
Milrinone: yes/none	0/0	0/0			
In stage III on admission	170	2			
IVIG: high/low/none	102/68/0	1/1/0	0.76		
Methylprednisolone: high/low/none	134/36/0	2/0/0	0.46		
Mechanical ventilation: yes/none	34/136	0/2	0.48		
Milrinone: yes/none	72/98	1/1	0.83		
In stage IV on admission	73	36			
IVIG: high/low/none	46/27/0	14/16/6	0.00	0.24	0.10–0.57
Methylprednisolone: high/low/none	40/33/0	17/15/4	0.11		
Mechanical ventilation: yes/none	72/1	19/17	0.00	0.01	0.00–0.10
Milrinone: yes/none	34/39	14/22	0.45		
All severe cases	599	38			

Our investigation found that 78% of fatalities before 2011 occurred on the way to or within 12 h after transfer to the designated hospitals.

### Prognosis Follow-Up

Eighteen children have been followed up by the end of December 2018. There were four patients with acute flaccid paralysis and three other ones with unilateral abducens nerve paralysis, and all of them had recovered within 2–5 months without further intervention. There were no CNS sequelae or any complications found for survivors who had received a large dose of methylprednisolone.

## Discussion

Our investigation shows that EV71 is the main virus that causes serious epidemics, severe case, and fatality of HFMD and that EV71 positive rate of severe HFMD cases had decreased coincident with the decrease of HFMD prevalence. Analyses suggest that elevated CRP and cTnI levels may be useful laboratory markers to identify severe HFMD cases at risk of systemic complications several hours before the onset of overt signs of deterioration. Hyperglycemia and leukocytosis are not risk factors for poor prognosis, which is inconsistent with the report from Hainan, China (14). Another study identified that being female and having light-reflex insensitivity, tachycardia, and high serum lactate levels are independent risk factors, and longer onset-to-hospitalization time is an independent protective factor for death in children with critical and severe HFMD (15).

Our investigation suggests that immediate IVIG and mechanical ventilation application to severe HFMD in early stage IV may improve pulmonary edema or hemorrhage and mortality. A protective-ventilation strategy might outperform common ventilation strategies in keeping normal blood pressure, but no fatality difference between ventilation strategies was found, which need more rigorous control studies to confirm. High airway pressure and PEEP may have extrapulmonary effects such as reducing the volume of venous return, increasing the afterload in the right atrium, decreasing cardiac output, decreasing visceral venous return, impairing renal function, and altering hormonal levels (13).

We found that milrinone is effective in reducing hypertension and sympathetic tachycardia and that methylprednisolone can decrease high body temperature, which has been proven by many cases, but neither of them contributed significantly to the survival rate in our data. In contrast, a study in Taiwan (China) found that a milrinone-treated group was associated with reduced mortality corresponding to attenuated sympathetic activity and cytokine production in comparison with a non-treated group (16). IVIG and methylprednisolone have been used in many countries and districts on a presumptive basis and have been shown to improve symptoms and decrease inflammatory factor storm for severe HFMD (17, 18). Elevated serum levels of inflammatory cytokines, including IL-3, IL-6, IL-12p40, and TNF-α, and decreased levels of serum biomarkers, including IL-1Ra, IL-8, IL-16, soluble ICAM-1, CXCL-1, and CCL27, were found in HFMD cases, which suggests that systemic inflammation is involved in the etiology of HFMD. In contrast, the associated biomarkers did not make any difference in the patients treated with methylprednisolone (19), and methylprednisolone was even associated with an increased risk of severe HFMD development (20). Therefore, further investigations are needed to determine the usefulness of steroid treatment for HFMD.

We also found that a large dose of IVIG did not outperform a low dose of IVIG in improving outcome of HFMD regardless of administration in any stage. There is no sufficient evidence to hasten the use of mechanical ventilation or IVIG for HFMD in stage II or III.

Persistent high fever, vomiting, lethargy, agitation, and irritability are indications of CNS involvement (21, 22). More specific neurological signs, such as myoclonic jerk (usually observed during the early stage of sleep but also seen in severe cases when patients are awake), truncal ataxia, and “wandering eyes” (rotary eye movement without fixation) are commonly observed in critical pediatric cases (23). We realize that it usually takes several hours for severe HFMD to deteriorate from stage III to stage IV, but there is usually <1 h to take quick action after it gets worse to stage IV. Severe cases should thus be closely monitored for signs indicative of CNS involvement, ANS dysregulation, and the development of cardiopulmonary failure; and timely intervention or even mechanical ventilation is the key to reducing fatality associated with severe HFMD.

The analysis shows that patients raised by grandparents contribute to fatality, which may be due to a delay in the early recognition of critical cases and a loss of prompt intervention. Therefore, in recent years, the health authorities require primary care physicians in villages or communities to pay more attention to these patients and emphasize frequent home visits.

Fatality of HFMD is the most common cause of medical disputes and medical compensation in China in recent years, which is the consensus of many pediatricians and lawyers in medical litigation. When we found that unsuitable transfer of critical patients may result in an increase in fatality and medical disputes, the health authorities had quickly equipped all departments of pediatrics with mechanical ventilators for all county-level and district hospitals and had held comprehensive training sessions on mechanical ventilation management. Health authorities emphasize contraindication to transfer and dispatch specialists to hospitals where there is no chance of transfer critical cases to the designated hospitals. HFMD fatalities had decreased significantly since the enforcement of new policies after 2011.

It is puzzling that the outbreaks of HFMD were so severe in Xiangyang, whereas adjacent areas were less impacted; and severe morbidity and mortality among districts, county-level cities, and counties were very different in Xiangyang. Another study also shows spatiotemporal differences in HFMD prevalence (24). We cannot convincingly explain the difference as the result of climate, harsh environment, insalubrity, large floating population, and/or poor education. In addition, there were no fatality in stage II and only two fatalities in stage III on admission in our data. When more severe cases and fatalities are enrolled and a randomized, double-blind, placebo-controlled trial design is adopted, we presume that the effects of IVIG and methylprednisolone administration in stages II or III may be confirmed and stage IV may be held at bay.

## Conclusions

The precise recognition of severe HFMD cases and prompt IVIG and mechanical ventilation application in early stage IV can significantly reduce fatality. Mechanical ventilation training programs and dispatch of specialists to hospitals where there is no chance of transferring critical HFMD cases to the designated hospitals are two key measures to reduce fatality.

## Data Availability Statement

The raw data supporting the conclusions of this article will be made available by the authors, without undue reservation.

## Ethics Statement

The studies involving human participants were reviewed and approved by the Ethics Committee of Baoan People's Hospital. Written informed consent from the participants' legal guardian/next of kin was not required to participate in this study in accordance with the national legislation and the institutional requirements.

## Author Contributions

All authors listed have made a substantial, direct and intellectual contribution to the work, and approved it for publication.

## Conflict of Interest

The authors declare that the research was conducted in the absence of any commercial or financial relationships that could be construed as a potential conflict of interest.

## Abbreviations

ANS: autonomic nervous system
CI: confidence interval
CNS: central nervous system
CA16: coxsackievirus A16
cTnI: cardiac troponin I
EV71: human enterovirus 71
HFMD: hand, foot, and mouth disease
PEEP: positive end-expiratory pressure
IVIG: intravenous immunoglobulin
MAP: mean airway pressure
OR: odds ratio.
